# Correlation between inflammatory factors and post-stroke pneumonia in diabetic patients

**DOI:** 10.3892/etm.2013.1103

**Published:** 2013-05-08

**Authors:** HONGMING ZHANG, XIAOYAN LI

**Affiliations:** Department of Cardiology, The General Hospital of Jinan Military Region, Jinan, Shandong 250031, P.R. China

**Keywords:** acute ischemic stroke, C-reactive protein, interleukin-6, pneumonia, diabetes mellitus

## Abstract

Pneumonia is the most common cause of mortality in stroke patients and it has been demonstrated to contribute to mortality and poor functional outcome following stroke in the majority of clinical studies. The risk of infection may be attributed to stroke-induced immunodepression syndrome (SIDS). Cytokine production is increased in SIDS. However, the correlation between biomarkers and the risk of post-stroke pneumonia in patients with diabetes mellitus is not clear. The aim of this study was to determine the correlation between pneumonia and the levels of C-reactive protein (CRP) and interleukin-6 (IL-6), as well as to identify early predictors of pneumonia in acute ischemic stroke patients with diabetes mellitus. Additionally, we investigated the impact of pneumonia on functional outcome after 1 month. A total of 106 ischemic stroke patients with diabetes mellitus who were admitted after the onset of symptoms were included in the study. They were divided into two groups, the pneumonia and non-pneumonia groups. CRP, IL-6, white blood cells (WBCs), mean body temperature and National Institutes of Health Stroke Scale (NIHSS) score were measured at the time of admission. The modified Rankin Scale score was assessed at 30 days. The levels of IL-6, CRP and WBCs, as well as mean body temperature were significantly higher in the patients with pneumonia than in the patients without pneumonia. There were also significant differences between the pneumonia and non-pneumonia groups in age, admission NIHSS score, length of hospital stay and dysphagia. Pneumonia patients had worse outcomes compared with patients without pneumonia at 1 month. Age, NIHSS score and dysphagia were significantly associated with pneumonia. WBCs and mean body temperature were not significant predictors of pneumonia. Older patients with more severe ischemic stroke are more susceptible to the development of pneumonia during the stay in hospital. Pneumonia contributes to poor functional outcome. IL-6, CRP, age, NIHSS score and dysphagia may predict the occurrence of pneumonia on the day of stroke symptom onset.

## Introduction

Pneumonia is a common cause of mortality in stroke patients (1–8). The risk of infection may be attributed to stroke-induced immunodepression syndrome (SIDS). SIDS is characterized by a loss of lymphocytes, cytokine production, reduced monocyte count and function, and interferon-γ deficiency. These effects are associated with pneumonia following stroke (9,10). Biomarkers may facilitate an early diagnosis of pneumonia in patients with acute ischemic stroke (11).

The correlation between biomarkers in acute ischemic stroke with diabetes mellitus, and the risk of pneumonia is not clear. The aim of this study was to determine the correlation between pneumonia and the levels of C-reactive protein (CRP) and interleukin-6 (IL-6), as well as to identify early predictors of pneumonia in acute ischemic stroke patients with diabetes mellitus and the effect of pneumonia on functional outcome after 1 month.

## Patients and methods

### Patients

Data for this study were obtained from the General Hospital of Jinan Military Region (Shandong, China). Records were collected from all ischemic stroke patients with diabetes mellitus from July 2009 to September 2012. Patients were excluded from the study if they: i) had an upper respiratory tract infection, ii) had a urinary system infection or iii) were immunocompromized by chemotherapy. Patients were defined as diabetic if they had known diabetes mellitus prior to the stroke, according to the diagnostic criteria of the Expert Committee on the Diagnosis and Classification of Diabetes Mellitus (12). The presence of pneumonia was determined using the Mann criteria of pneumonia (13). The study was approved by the ethics committee of the General Hospital of Jinan Military Region and written informed consent was obtained from each of the participants.

### Assessment

At admission, a computed tomography (CT) scan was performed to rule out hemorrhage. White blood cell (WBC), CRP and IL-6 levels were measured on day 1 along with the mean body temperature. The IL-6 and CRP levels were measured by chemical luminescence immunoassay and WBC levels by electrical impedance.

The National Institutes of Health Stroke Scale (NIHSS) score of each patient was assessed by a neurologist. Functional outcome was assessed at 30 days using the modified Rankin Scale (mRS). Poor outcome was defined as mortality or moderate or severe disability (mRS score, 3–6).

### Statistical analysis

Differences in patient characteristics and clinical variables in the pneumonia and non-pneumonia groups were analyzed using the χ^2^ test and t-test for continuous variables, respectively. The logistic multiple regression model was used to analyze risk factors and pneumonia. Data analyses were performed with commercially available statistical software (SPSS version 11.0; SPSS, Inc., Chicago, IL, USA). P<0.05 was considered to indicate a statistically significant difference.

## Results

The clinical characteristics of stroke patients with and without pneumonia are compared in Table I. Patients with pneumonia were significantly older than those without pneumonia. There were also significant differences between the pneumonia and non-pneumonia groups in admission NIHSS score, length of hospital stay and dysphagia. The rates of hypertension, metabolic syndrome and coronary artery disease were, however, similar in the two groups.

The serum levels of IL-6, CRP and WBCs, as well as mean body temperature in the patients with and without pneumonia are presented in Table II. The levels of IL-6, CRP and WBCs, and the mean body temperature were significantly higher in the pneumonia group than in the non-pneumonia group.

The functional outcomes of the ischemic stroke patients with and without pneumonia after 1 month are shown in Table III. Pneumonia patients had worse outcomes than patients without pneumonia at 1 month. There were more patients with severe disability (mRS score, 3–6) in the pneumonia group than in the non-pneumonia group.

The risks for pneumonia are shown in Table IV. Age, NIHSS score, IL-6, CRP and dysphagia were significantly associated with pneumonia. WBC level and mean body temperature were not significant predictors of pneumonia.

## Discussion

The rate of pneumonia in this study was 30.2%, which is higher than that reported by previous studies (14–17). Since the subjects were all ischemic stroke patients with diabetes mellitus, this may indicate that subjects with diabetes are more prone to infection. However, a patient sample size of 106 may have been too small to identify a reliable rate of pneumonia.

The current study demonstrates the increased risk of pneumonia following ischemic infarction for older patients with a more severe stroke. Pneumonia increases the length of hospital stay. IL-6 and CRP levels were significantly associated with pneumonia. Additionally, pneumonia had a detrimental impact on outcome 1 month after stroke.

Previous studies have examined the risk factors for infection (5,18–22) These risk factors include greater stroke severity on admission, total anterior circulation infarction, Barthel Index <5, Glasgow Coma Scale score <9, infarction in the territory of the middle cerebral artery, increased age, current atrial fibrillation, tube feeding and a history of congestive heart failure. Amelioration of neurological impairment and prevention of complications are major goals of stroke treatment. However, conservative measures provide limited protection against post-stroke pneumonia; this raises the possibility that stroke-induced alteration of the systemic immune response is significant.

The current study shows that the risk factors for pneumonia are age, NIHSS, dysphagia, IL-6 and CRP. SIDS may be responsible for the increases in the levels of IL-6 and CRP. Therefore, modulating cytokines and the immune system may be a new treatment strategy for acute ischemic stroke with pneumonia (23).

The correlation between infection risk and more severe ischemic infarctions defined by higher NIHSS score on admission has been established (24–27). Several randomized trials have demonstrated a reduction of the infection rate in stroke patients by prophylactic administration of antibiotics (28–31). The early initiation of antibiotic treatment in older patients with severe ischemic stroke should be considered to improve the clinical outcome.

In conclusion, patients with more severe ischemic stroke and increased age are more susceptible to the development of pneumonia during their stay in hospital. Pneumonia contributes to poor functional outcome. IL-6, CRP, age, NIHSS score and dysphagia may be predictive factors for the occurrence of pneumonia on the day of stroke symptom onset.

## Figures and Tables

**Table I.** Baseline characteristics of the study population.

Characteristic	Non-pneumonia group (n=74)	Pneumonia group (n=32)	P-value
Age (years)	68.85±2.40	73.71±6.30	<0.0001
Hypertension	23	18	NS
Metabolic syndrome	16	10	NS
Coronary artery disease	15	11	NS
Admission NIHSS score	9.6±0.7	14.50±0.6	<0.0001
Dysphagia	9	11	<0.0001
Length of hospital stay (days)	10.2±0.5	19.3±3.2	<0.0001

Data presented are mean ± SD. NIHSS, National Institutes of Health Stroke Scale; NS, no significance.

**Table II.** Markers of infection.

Marker	Non-pneumonia group	Pneumonia group	P-value
Interleukin-6 (pg/ml)	9.2±1.6	27.1±4.3	<0.0001
C-reactive protein (mg/l)	13.4±3.5	48.4±10.4	<0.0001
White blood cells (Gpt/l)	8.3±0.8	12.9±1.7	0.034
Mean temperature (°C)	36.7±0.1	38.4±0.2	<0.0001

Data presented are mean ± SD. Gpt, ×10^9^.

**Table III.** Functional outcome at 30 days.

mRS score	Non-pneumonia group	Pneumonia group	P-value
0–2	48	7	<0.0001
3–6	24	25	<0.0001

mRS, modified Rankin Scale.

**Table IV.** Predictors for pneumonia.

Variable	OR (95% CI)	P-value
Age (years)	1.66 (1.11–1.81)	<0.021
NIHSS score	1.36 (1.14–1.62)	<0.0001
C-reactive protein (mg/l)	1.54 (1.22–1.73)	<0.042
Interleukin-6 (pg/ml)	36.6 (2.7–582.6)	<0.001
White blood cells (Gpt/l)	26.5 (1.9–362.0)	>0.05
Mean temperature (°C)	21.2 (2.4–221.3)	>0.05

OR, odds ratio; CI, confidence interval; NIHSS, National Institutes of Health Stroke Scale.
